# Perceptions of labour pain management of Dutch primary care midwives: a focus group study

**DOI:** 10.1186/s12884-015-0795-6

**Published:** 2016-01-16

**Authors:** Trudy Klomp, Ank de Jonge, Eileen K. Hutton, Suzanne Hers, Antoine L. M. Lagro-Janssen

**Affiliations:** Department of Midwifery Science, AVAG and EMGO Institute for Health and Care Research, VU University Medical Center Amsterdam, Van der Boechorststraat 7, D4-40, 1081 BT Amsterdam, The Netherlands; Department of Primary Care and Community Care, Women’s Studies Medicine, Radboud University Medical Centre Nijmegen, Nijmegen, The Netherlands; Midwifery Education Program, McMaster University Hamilton, Hamilton, ON Canada

**Keywords:** Midwifery, Midwife-led care, Labour pain, Pain management, Childbirth

## Abstract

**Background:**

Labour pain is a major concern for women, their partners and maternity health care professionals. However, little is known about Dutch midwives’ perceptions of working with women experiencing labour pain. The aim of this study was to explore midwives’ perceptions of supporting women in dealing with pain during labour.

**Methods:**

We conducted a qualitative focus group study with four focus groups, including a total of 23 midwives from 23 midwifery practices across the country. Purposive sampling was used to select the practices. The constant comparison method of Glaser and Straus (1967, ren. 1995) was used to gain an understanding of midwives’ perceptions regarding labour pain management.

**Results:**

We found two main themes. The first theme concerned the midwives’ experienced professional role conflict, which was reflected in their approach of labour pain management along a spectrum from “working with pain” to a “pain relief” approach. The second theme identified situational factors, including time constraints; discontinuity of care; role of the partner; and various cultural influences, that altered the context in which care was provided and how midwives saw their professional role.

**Conclusion:**

Midwives felt challenged by the need to balance their professional attitude towards normal birth and labour pain, which favours working with pain, with the shift in society towards a wider acceptance of pharmacological pain management during labour. This shift compelled them to redefine their professional identity.

**Electronic supplementary material:**

The online version of this article (doi:10.1186/s12884-015-0795-6) contains supplementary material, which is available to authorized users.

## Background

Labour pain is a major concern for women, their partners, and maternity health care professionals and has received intense coverage in the Dutch nationwide media. Although the Netherlands has a tradition of birthing without the use of pharmacological pain management, such as epidural analgesia, the number of Dutch women using epidural analgesia has risen over the past decade [1, 2]. In 2012, 17.6 % of women without a planned caesarean section used epidural analgesia compared with 5.4 and 11.3 % in 2003 and 2008, respectively. The Netherlands has a community-based maternity care system, with approximately 84 % of all pregnant women starting antenatal care in midwife-led care and around 55 % of women starting their labour in midwife-led care [1]. Women with low-risk pregnancies undergoing midwife-led care can choose to give birth either at home, in a birth centre or in a hospital at the onset of labour while being attended by their own midwife. If risk factors or complications arise, women are referred to obstetrician-led care. Medical interventions such as pain medication during labour, epidural analgesia, continuous foetal monitoring and induction or augmentation of labour only take place in obstetrician-led care in hospital settings. In these situations, community midwives transfer care to the responsibility of an obstetrician. Typically after the transfer of care from community midwives, midwives working in a hospital setting take over care under the supervision of obstetricians. If women indicate during their pregnancy that they want to undergo analgesia with pharmacological agents, including epidural or other pain medication, they may have a consultation with an obstetrician, but most of them will start their labour in midwife-led care. As soon as they are in active labour and still choose to undergo pharmacological pain relief, the midwife will refer them to an obstetrician in the hospital setting. In this situation, midwives will experience no or hardly any implication in terms of diminishing caseloads or financial penalty.

Dutch midwives emphasise that labour is a physiological process and that labour pain may be a significant factor in the empowerment of women and in their relationship with their newborn babies [3]. This concept is in line with a specific cultural perception among Dutch women that is connected with the concept of natural childbirth [4]. Leap introduced two distinct approaches to labour pain that tend to be adopted by health professionals [5, 6]. The first is the “working with pain approach”, which involves providing women with support to help them cope with labour pain. The second is the “pain relief” approach, which involves offering pharmacological management to women in labour in order to minimise labour pain.

In 2008, a Dutch guideline concerning pain medication (or an epidural) was introduced [7]. This guideline states that a woman’s request for pain medication during labour is, of itself, an adequate medical indication to provide pain relief. It also states that epidural analgesia should be the method of choice for the elimination of labour pain. The guideline has changed attitudes towards labour pain management of women, their partners and maternity health care professionals in the Netherlands [1, 2]. Midwives, too, may have changed their perception, possibly shifting from the traditional “working with pain” approach towards a “pain relief” approach. However, to the best of our knowledge, there have been no previous studies of Dutch midwives’ perceptions regarding working with women undergoing labour pain. The aim of this study was to explore primary care midwives’ experiences while providing support to women undergoing labour pain and to determine whether midwives consider that their own attitude towards labour pain has changed in response to the changing attitudes towards this topic in society.

## Methods

### Ethical approval

Our study was approved by the Medical Ethical Committee of VU University Medical Center (VUmc) in Amsterdam. All midwives gave written informed consent prior to participation in the study.

### Design

We used a qualitative design and conducted four focus groups with a total of 23 midwives from 23 midwifery practices across the Netherlands.

### Data collection

A total of 26 practices across the country were asked to participate in the focus groups. Three declined because of time constraints.

### Procedure

Participating midwives were self-selected from within each practice. We included only one midwife per practice, to obtain a broad view of midwives’ attitudes. The decision to use focus groups was based on the fact that professionals are better able to share knowledge in social interactions. It was also thought that this approach might elucidate details of professional values and culture [8]. We used purposive sampling, and the selected practices were located in various parts of the country, in rural, rural/urban, and urban areas. We included midwives who worked in solo, duo, and group practices. The researcher informed each practice and each midwife that any information obtained in the focus group discussion would be handled confidentially. The discussions were taped using a digital voice recorder. The first author kept field notes in a logbook, giving details of the context of the discussion, conditions in the focus group and reflections while carrying out her own role as an interviewer.

Midwives were asked to fill in a form containing questions about their personal characteristics. All focus group proceedings were conducted face-to-face in Dutch by the first author (TK) and one trained midwifery student (SH) as moderator and co-moderator. Midwifery students were involved in the study as part of their midwifery research education programme. They signed a confidentiality statement before they were involved in the study. Some midwifery students acted as hostesses while others audiotaped the group discussion. The focus groups were conducted in a range of settings, such as midwives’ practices, a cultural centre, and meeting rooms both at the university and at a midwifery school. The intention was to continue with the focus groups until we reached data saturation.

### Focus group topic list

Focus group discussions were guided by a topic list. The questions were not formally phrased but key topics were formulated to help the moderator structure the discussions (Additional file 1). These key topics were selected on the basis of input related to the two approaches to women in labour pain formulated by Leap [5, 6]. The opening question was:

*“We are interested in your perception towards working with women who are experiencing labour pain. What do you think is the best way to help women in labour with their labour pain?”*

### Analyses

All of the discussions were transcribed by three midwifery student researchers. In addition, the constant comparison method was used to elucidate midwives’ perceptions of labour pain management [9, 10]. The micro-analytic process of developing concepts from the data involved repeated reviews of the transcripts (both written and taped) by TK, assisted by two midwifery student researchers. The transcripts were coded by the researchers (TK, SH and SB). Data were analysed and discussed at regular meetings attended by all the researchers, by which repetitive ideas, similarities, and differences were identified. Events that shared common characteristics were cross-linked, while key phrases were identified and coded. The analyses generated new information that was explored in subsequent discussions [8, 11].

The iterative process of constantly comparing text fragments enabled any research bias to be minimised. We used memo writing to extract explanations from the data (10). During regular research team meetings, we discussed and explored theories in order to ensure the validity and accuracy of our analysis.

The main themes that we identified are discussed below. These are illustrated by quotes from the Dutch verbatim transcript, translated into English by a professional translator. The following details were added to the quotes: participant number (Px); explanation added by authors is in square brackets []; and omitted text, indicted by […].

## Results

We held four focus groups between June 2011 and July 2012. Midwives ranged in age from 24 to 56 years. They came from various types of practices (two solo, five duo and 15 group practices) located in areas with varying degrees of urbanisation (nine urban, seven urban/rural and six rural areas). The length of midwives’ experience ranged from 1 to 35 years. The characteristics of all participants are outlined in Table 1. Each focus group session lasted approximately 90 min.

**Table 1:** Characteristics of focus group participants

	Participating midwife no. (Px)	Age (yrs.) from-to	Number of practice organisation solo/duo/group (x midwives)	Midwifery experience in years from–to
F1	P1–6	24–52	2 solo	1–30
			1 duo	
			1 group (3)	
			2 group (4)	
F2	P7–12	24–56	2 duo	5–35
			1 group (3)	
			2 group (4)	
			1 group (5)	
F3	P13–17	27–50	2 duo	3–23
			2 group (4)	
			1 group (5)	
F4	P18–23	29–56	1 duo	5–24
			2 group (3)	
			1 group (4)	
			1 group (5)	
			1 group (6)	

The following two overarching themes emerged from the analysis:

1. Midwives’ experienced professional role conflict, which was reflected in their approaches to labour pain. These approaches were bound within midwifery care and connected with the predominate beliefs of natural childbirth.
2. Midwives’ perception of that their professional role was influenced by situational factors, including time constraints; discontinuity of care; role of the partner; and various cultural influences.

## Midwives’ professional role conflict

### Perceptions of the ‘working with pain’ and ‘pain relief’ approaches

Midwives whose approach was to help women work with pain described childbirth as a natural biological process. They believed that important birth hormones are released, which allow women to manage labour pain without the need of pharmacological pain management, including the use of epidural analgesia. Midwives described these hormones as essential for labour pain management, for allowing labour pain to be tolerable for women, for mother-child bonding, and for women’s self-esteem. This is exemplified by the following quotes:

*“Pain is an essential part of the labour process […]. Your body will release those endorphins and these [hormones] will influence mother and child bonding … will influence the awareness of pain. Overall, moments of pain are potential opportunities for inner growth. If women are supported in their labour pain, I believe, that this can be very important for them. We have to consider very carefully whether we should sedate all those important moments in women’s lives…… (*P5).”

*“As if pain medication is the solution to everything* (P7).”

For most midwives in our study, “working with pain” was seen as preferable to providing pain medication or an epidural to women. Conversely, these midwives faced the inherent uncertainty of childbirth, not knowing how long labour will last nor how well an individual woman might be able to tolerate labour pain.

*“… It is very hard to assess [intensity of labour pain]; … this is manageable and this not… With one woman you feel ‘this is horrible’ but she says ‘well it was all right actually’. For another you think that ‘she will manage’ yet she perceives labour as hellish…..* (P16).”

At the same time, some midwives said that their perceptions of pain medication and epidurals had changed in the years since the introduction of the pharmacological pain relief guideline in 2008 [6]. This was because of women’s changed attitudes toward labour pain, which in turn was reflected by the way midwives supported women in labour pain. Midwives stated that they had shifted towards a more pain-relief oriented approach than the one they applied before the introduction of the guideline.

“……*the client has already made a personal decision to use pain medication, so I just arrange this for her … this is really different compared with 10 years ago…* (P3).”

All the midwives in our study were happy with the availability of pharmacological agents for pain management, including epidural analgesia, for women who need it, such as those who have been traumatised by previous childbirth experiences of labour pain or those whose labour do not progress. Nevertheless most midwives believed that they play an important role in helping women manage their labour pain without the use of pain medication or epidural analgesia.

*“Above all, it is fantastic that pain medication exists and that it is relatively easy to obtain if a woman really wants to use it, but our attitude also has a big effect. If we also take the view that labour pain is quite normal, this will make it more manageable for women* (P5).”

Midwives said that they would like to have more influence on the process of labour pain management. Some said that women do not always accept the supporting role of a midwife because their assessment of their own ability to work with pain differs from that of the midwife.

*“[…] this is a woman who I could have supported through labour pain but she decided to have pain medication and I find that difficult. At that point I think ‘If we could have waited for just one more hour then she would have been fine’, but yes, women are no longer prepared to accept ‘just one more hour’……* (P21).”

Another factor that worried midwives seemed to be the prevalence of the ‘pain relief’ view, in the media and among women, their partners, and maternity health care professionals, resulting in an excessively low threshold for the provision of pain medication or an epidural. In a situation where the support of women in labour seems to be losing ground to pain medication or use of epidural analgesia, as the standard approach to labour pain, midwives felt that they were no longer able to use their training in midwifery standards to provide such support.

*“Pain medication seems to be a substitute for coaching women in labour pain, I believe this development is a major cause of concern* (P4).”

*“I caught myself thinking very unkind thoughts: ‘what a fussy woman you are, everything has to be totally organised, you don’t want any pain, you don’t want to breastfeed… only then do you want a child!* (P14).”

However, most midwives in our study expressed the view that facilitating women’s satisfaction with the childbirth experience was the most important aspect of labour pain management, overriding their own beliefs about normal labour. They seemed to feel that pain relief during labour should be seen as a spectrum of pain management, and not as a simple dichotomous choice.

*“When they have given birth, I know that it is incredibly important for women to be able to look back at a satisfying birth process rather than thinking ‘that was sheer hell’. I’d be the first one to approve pain relief, and to offer effective counselling and consultation* (P21).”

Midwives in our study realised that the world is changing. With these changes, and women being more outspoken now, they may request medical interventions even before they are actually in labour.

*“We have to keep up with the times and admit that women now have access to various forms of labour pain medication. It’s a sign of prosperity to be able to give birth with less labour pain or none at all* (P22).”

Nevertheless, according to the midwives, most women prefer to undergo natural labour without pain medication or epidural analgesia if possible, but they feel comfort and assurance in knowing that it is available if they should need it during labour. They expressed the opinion that, once the methods of pain relief available in the region have been explained to them, women have enough confidence to start their labour process without fear.

*“Women often tell us: ‘These midwives won’t be difficult when it comes to pain medication. I know it [pain medication] is available, I just want to try and see how far I can go without it* (P3).”

## Situational context

Midwives mentioned that factors such as time constraints, discontinuity of care, the role played by the partner and cultural influences influenced their perception of their professional role in helping women with labour pain.

### Time constraints

Midwives identified time constraints, in both the antenatal and labour care, that limit their ability to engage with women in relation to ‘working with’ labour pain. This limited time availability seemed to be a major constraint to providing continuous support for women in labour. Midwives believed that having more time to provide continuous support in labour would increase their fulfilment in their work and would be more beneficial to the women they support and care for. They suggested that women might need less pain medication or epidural analgesia if midwives provided continuous support to women in labour.

“*…What I have to offer to women in labour, how I might empower women to embrace the birth experience, but I would like to give more, in terms of hours, in being there with women….after all you know this will give you more energy* (P9).”

Most midwives expressed the importance of providing information and antenatal counselling about the labour process itself, as well as the importance of managing labour pain. They wanted to spend more time having full and frank discussions with women about labour, the different possible outcomes, and the fact that labour will always be unpredictable.

*“It is important to provide a good explanation [during pregnancy], to give more details about the nature of an epidural and to describe remifentanil, as well as the pros and cons. However, you also need to tell them that things often do not turn out the way you might expect. Now you [women] might think: ‘no epidural for me, but when it comes right down to it you might want one ......Discussing all these issues takes time’* (P1).”

### Discontinuity of care

Most of the midwives in our study felt dissatisfied about not being able to provide continuity of care when women with labour pain were transferred to secondary obstetric care to receive pain medication or epidural analgesia. They stated that they would like to provide continuous support for women in labour, regardless of whether or not the woman under their care needed to be transferred to obstetrician-led care; they want to continue to provide their midwifery care for such women.

*“When we arrived at the hospital, they [hospital staff] thought she was not in active labour [subsequently, the midwife went home and left the woman and her partner to her colleagues in hospital] Two hours later, she was eight centimetres’ dilated, and it was too late for pain medication..... For the woman in question, those two lonely hours [as there was nobody to support her] were very traumatic. I told the physician on the phone ‘I think that things will go very quickly, but afterwards I felt that I should have done more. I was very dissatisfied with the way things went. There was no acknowledgement of that pain, which turned out to be very crucial. I should have stayed with them in the hospital* (P5).”

### Partner’s role

Most midwives expressed the belief that partners play a vital role in labour pain management. A major factor in the midwives’ view of their supporting role was their commitment to the partners. This involved informing them about the labour process and involving them in the process itself.

*“Some partners are well aware of the contribution they can make. When a partner feels that he is being useful, that he can make a genuine contribution, then he can make all the difference. That will really help a woman in labour. I also ask them ‘What is your role in this?’ ‘What do you think about it?’* (P21)*”.*

Midwives stated that they aim to give partners the same information that they give pregnant women themselves in order to strengthen the important supporting role played by partners during labour.

*“I will always involve the partner; I will discuss the nature of the labour pain at that time with both of them. But sometimes, when a woman is in active labour and she is struggling with labour pain, the partner will say: ‘breathe, breathe, breathe’, followed by: ‘this is really too much for my wife, how much pain can she take?!’ It is, of course, very difficult to see your much loved wife suffering from labour pain. I then explain the nature of the labour process to him: whether what is happening is still normal or not*…(P12).”

### Cultural influences

Some midwives pointed out that they also have to deal with specific cultural beliefs about labour pain management. They seemed to be aware of the need for a diverse range of support skills in order to help women manage their labour pain.

*“To some extent, I also believe that this is culturally determined, e.g. in other cultures women tend to make a lot of noise during labour. The idea is that the more pain they feel, the more respect they will get from their husbands. And some of these women are just very happy that we have pain relief here, because they don’t have that at home (P5).”*

At the same time, midwives were aware that women from other parts of the world might have quite a different approach to labour pain.

*“We also have a very special group of mostly parous women from Africa. These women have given birth before, in Africa, without pain relief. They just give birth without [pain medication], they don’t even question it. They do not see this as an issue, it is just something you do (P6, midwives nodded their head and smiled).”*

Midwives who work in the religious region of the Netherlands told us about their experiences with women in labour pain.

*“In our region, people believe what is written in the Bible: ‘Thou shalt give birth in grief’. This is a belief that I share. For this reason, in our practice, many women do not want to use pain relief, and they wonder ‘is it permissible to use labour pain medication?’ We have many such women…..* (P1).”

## Discussion

Our results revealed two main themes: 1] midwives’ experienced professional role conflict, which was reflected in their approach of labour pain management along a spectrum from “working with pain” to a “pain relief” approach; 2] situational factors, including time constraints; discontinuity of care; role of the partner; and various cultural influences, that altered the context in which care was provided and how midwives saw their professional role.

Most midwives had been trained to promote natural childbirth [12, 13], and they firmly believed in this approach. A number of them were experiencing something of a professional identity crisis, stating that some women are no longer prepared to accept professional midwifery care during labour. At the same time, most of the midwives in our study thought that they were more prepared to arrange for pain relief nowadays compared with 10 years ago [1, 2]. This has implications for midwives’ daily practice in supporting women undergoing labour pain. Pain medication or use of epidural analgesia during labour are only provided in hospital maternity units following transfer to obstetrician-led care. Accordingly, requests for pain medication or an epidural results in a discontinuity of care in the Netherlands. This discontinuity of care was a cause of frustration and dissatisfaction for the midwives. Because of time constraints within the maternity care model in which Dutch community midwives work, midwives seemed unable to provide adequate continuity of care. They believed this leads to a situation in which some of the women requesting pain medication or epidural analgesia do not really want it, and alternatively, some who want pain medication do not actually obtain it. Compared with other countries where midwives work autonomously, Dutch midwives have a relatively high caseload, which makes it difficult for them to stay with women in early labour after referral to obstetrician-led care [14]. Research has shown that continuity of care in labour with a known midwife, who has built a trusting relationship during pregnancy, can reduce interventions and the use of epidurals, thereby leading to an increased number of spontaneous vaginal births and maternal satisfaction [15, 16]. Dutch women experience a relatively low intervention rate during labour [1, 3, 17]. This may have been attributed to the high degree of continuity of care when women received midwife-led care at home, in maternity care units in hospitals, or birth centres. With the increasing numbers of Dutch woman who are transferred to obstetrician-led care during labour [1, 17], it is possible that interventions will increase, with a potential decrease in maternal satisfaction. Paradoxically, this is in contrast with the intention of legislation on access to pain medication (or epidural analgesia), that aimed to increase women’s satisfaction with their experience of labour [7].

Other study results confirm that midwives obtain genuine job satisfaction from providing continuity of care and from the relationships that they establish with the women in their care [18, 19]. These findings also show that midwives feel frustrated when they are unable to practise midwifery care in a way that conforms to their view of normal birth [19]. Midwives experienced this role conflict in the context of the change in maternity care approach towards one that is increasingly obstetrically dominated and that is reflected in a technocratic paradigm that emphasises mind-body separation [20]. Midwives’ approaches to maternity care are more embedded in a humanistic and holistic approach. These two approaches emphasise the mind-body connection and coherence of body, mind and soul [20–23]. Although the Netherlands has had the reputation of upholding physiological birth through a strong midwifery approach, there appears to be a shift towards an obstetrically dominated system [17]. This is apparent in the findings of perceptions of midwives’ approaches towards labour pain in our study. One could argue that in combining components of all three paradigms, mind, body and soul, one could attain the most effective maternity care system for mothers and their infants [20]. One should bear in mind in our analyses regarding the role of pain in labour that the approaches of health care professionals towards labour pain are always a creation of their individual ‘bodies, minds and cultures’ [6, 24, 25].

Some literature reviews have suggested that having ‘continuity of carer’ during pregnancy and labour was less important for women in labour than it was for their supporting midwives [26, 27]. It seemed more important for women to receive consistent care from health care professionals whom they trusted. Another study published after 2000 reported the opposite findings: continuity of carer is important to women and increases maternal satisfaction with their childbirth experience [15, 28].

Midwives in our study expressed their frustration at having to transfer their patients to obstetrician-led care for pain medication or epidural analgesia. Hyde and Roche-Reid [29] concluded that modernity has implications for the role of the midwife. They found that midwives believe their role is to empower women and to facilitate choices for women through dialogue. Midwives in our study also believed in the value of maintaining a dialogue with their clients and with their clients’ partners.

We found that pain medication or use of epidural analgesia during labour was not really an issue for those midwives who worked in a particularly religious region of the Netherlands and those who attended women from African countries. Our findings are consistent with those of Callister et al. [30], who found that women’s perception of pain and their pain behaviour are culturally determined. This research described the cross-cultural appreciation of empowerment of women by the challenge of labour. Women might feel empowered by dealing with labour pain, which results in creating new life [31]. Culturally diverse women who have support from an unknown birth attendant and give birth in a technologic environment with routine interventions are more likely to experience anxiety and labour pain [32]. We are not aware of any recently related studies of attitudes to pain in labour of women with diverse religious backgrounds.

Midwives in our study were aware that the ability to work with labour pain is more important, in terms of a woman’s satisfaction with her childbirth experience, than actual avoidance of labour pain [33] or receiving pain medication or epidural analgesia [34]. However, midwives felt there was a shift in terms of women wanting better access to pain medication or epidural analgesia. Midwives knew that evidence showed that the use of pharmacological pain relief and epidurals is not associated with a positive experience of childbirth [34]. In most cases, midwives viewed themselves as being sufficiently experienced and well equipped to support women in labour. They suggested that most women feel reassurance in knowing that pain medication or epidural analgesia is available if they need it, but that they prefer to experience childbirth without it if possible. Midwives in this study wanted to spend time providing balanced information and counselling in the antenatal period, as well as sufficient time to support women in labour pain. They felt that most women were prepared to rely on their midwife’s expertise to support them through labour pain. This finding is consistent with other studies about shared decision making [35, 36] that underline the importance of respectful listening and open communication in building good relationships between women and the health care professionals who provide care for them. At the same time, midwives complained about having too little time to carry out their full range of duties effectively.

Midwives in our study believed that most partners played a crucial role in the management of women’s labour pain. This is supported by other studies who found that, when the birthing partner of women provided them with support and encouragement in the use of pain control techniques (breathing, massage, distraction), women were less likely to ask for epidural analgesia [37, 38]. Midwives also pointed out that, during the labour process in which they are unable to encourage women’s partners, some partners might react with expressions of helplessness. It is for this reason that midwives informed women in labour and their partners about the process itself, and about how they might best manage the process and pain together [39]. A study in Italy showed that men might be affected by a dominant culture of pain medication. In this study men’s experience of childbirth was improved when their partners used epidurals [40]. These men experienced less anxiety and stress and felt more involvement, participation and satisfaction with the experience of childbirth. Dutch men might also be influenced by this phenomenon, but further research is needed to explore experiences of partners of women in labour regarding management of labour pain.

### Limitations

This study had several limitations. Our sample size of four focus groups was relatively small. However, the use of a robust sampling frame made it possible to capture a wide range of perceptions from midwives with varying amounts of experience, practising in a variety of clinical settings. In this study, we achieved data saturation with four focus groups. The fact that the interviewer was a former midwife might be another limitation, as the peers involved may have given the answers that they thought the interviewer wanted to hear. However, given the wide variety of perceptions captured, we believe that this had no significant influence in the results of this study.

### Strengths

Our study had several strengths. To our knowledge, this is the first study to evaluate the perceptions of primary care midwives in the Netherlands regarding the management of labour pain, and to determine whether midwives think their perceptions have changed in response to the changing attitudes in society towards labour pain.

## Conclusions

Midwives felt challenged by the need to balance their professional attitude towards normal birth and working with labour pain with the shift in society towards a wider acceptance of pain medication and use of epidural analgesia during labour. Most midwives in our study believed that the issue of pain relief is not a simple dichotomous choice, but rather that it should be seen as a spectrum of labour pain management. At the same time, their perceptions seem to have shifted: now midwives are more prepared to offer pain relief compared with 10 years ago. Therefore, midwives felt compelled to redefine their professional identity.

## Additional file

Additional file 1:
**Topic list** (DOCX 15 kb)
