# Supplementary material for: Perceptions of labour pain management of Dutch primary care midwives: a focus group study
Source: BMC Pregnancy Childbirth. 2016 Jan 16;16:6. doi: 10.1186/s12884-015-0795-6 (PMC4715289; doi:10.1186/s12884-015-0795-6)
Supplement: Additional file 1: — Topic list (DOCX 15 kb) [file 12884_2015_795_MOESM1_ESM.docx]

Additional file 1.Topic list

Perceptions of labour pain management of Dutch primary care midwives, a focus group study


Opening question:
*“We are interested in your perception towards working with women who are experiencing labour pain. What do you think is the best way to help women in labour with their labour pain?”*


Probes:

- Perception of labour pain?
- Role of the midwife?
- Role of women’s partner?
- Role of implementation of the CBO guideline ‘Medicinal Pain Relief’ in 2008-2009?
- Possibilities/availabilities at home?
- Referral for medicinal pain relief (when; women’s involvement in decision making; partner’s role)?
